# The protocol for a randomised-controlled trial of the evaluation of the tolerance and safety of early enteral nutrition in children after percutaneous endoscopic gastrostomy placement. (protocol version 09.01.2015)

**DOI:** 10.1186/s12887-016-0705-8

**Published:** 2016-10-07

**Authors:** Anna Wiernicka, Małgorzata Matuszczyk, Agnieszka Szlagatys-Sidorkiewicz, Ewa Toporowska-Kowalska, Katarzyna Popińska, Urszula Chlebowczyk-Grzybowska, Ewa Hapyn, Jarosław Kierkuś

**Affiliations:** 1 Department of Gastroenterology, Hepatology and Feeding Disorders, The Children’s Memorial Health Institute, Aleja Dzieci Polskich 20, 04-730 Warsaw, Poland; 2 Department of Pediatrics, Gastroenterology, Hepatology and Nutrition, Medical University of Gdańsk, Gdańsk, Poland; 3 Department of Alergology, Gastroenterology and Nutrition, Medical University, Łódź, Poland; 4 Department of Pediatrics, Nutrition and Metabolic Disorders, The Children’s Memorial Health Institute, Warsaw, Poland; 5 Department of Pediatrics, Medical University of Silesia, Katowice, Poland; 6 Department of Pediatrics and Gastroenterology, Area Hospital in Toruń, Toruń, Poland

**Keywords:** Early enteral nutrition, Children, Percutaneous endoscopic gastrostomy placement

## Abstract

**Background:**

The appropriate time to initiate enteral nutrition after the placement of a percutaneous endoscopic gastrostomy (PEG) tube has been an area of limited research. There are no sufficient randomised prospective controlled trials in the paediatric population comparing the safety and tolerance of early feeding (3 h) after PEG placement. In order to reduce the period of fasting, inadequate nutritional support, and hospitalisation time, we decided to devise this study.

**Methods/Design:**

This study is a multicentre, randomised, open-label trial designed to evaluate the tolerance and safety of early enteral nutrition after PEG placement in children. Patients are randomised to receive the first feeding bolus with a polymeric diet (1 kcal/ml) via a feeding tube 3 h after the PEG placement (group I - early enteral feeding) or 8 h after the procedure (group II - late enteral feeding). The key objective of the study is to compare the tolerance and safety of the early- and late-feeding modes after PEG placement in children. The primary endpoint is the number of patients who will achieve full feed (total fluid and caloric requirements) within 48 h of the first feeding bolus. The secondary endpoints are: the number of early and late complications, the duration of hospitalisation after PEG placement, gastric residuals (ml) total in the period up to 48 h since the first feeding bolus.

**Discussion:**

To our knowledge this is the first study in paediatric patients to evaluate the tolerance and safety of early enteral nutrition after PEG placement. The goal is to establish an optimum standard procedure in the group of paediatric patients qualified for the PEG insertion procedure in Poland.

**Trial registration:**

ClinicalTrials.gov ID NCT02777541, registration date 05/18/2016.

## Background

The percutaneous endoscopic gastrostomy (PEG) technique for gastrostomy placement was developed as an alternative to surgical placement with the advantage of faster recovery. Although the benefits and techniques for the insertion of PEG have been described and accepted, feeding after PEG placement is not as clear. Traditionally, tube feedings have been delayed after PEG placement to the next day and up to 24 h postprocedure. This decision is based on a standard convention, extrapolated from surgical guidelines, with little data to support the withholding of feedings after PEG placement. Over the past 15 years, many studies have examined the use of early PEG feedings after insertion. Results from various randomised controlled trials (RCTs), mostly in adults, indicate that earlier feeding might be an option. In some adult centres, it is reported that the procedure can be performed in the morning and the patient discharged the same day. Despite the recent literature indicating early PEG feedings as a safe alternative, the common practice continues to be to significantly delay post-PEG feedings. It is essential, especially in children, to reduce to a minimum fasting time and begin to improve nutritional status as soon as possible. In view of the financial pressures on the healthcare system, the economic aspect is also essential. A short stay in hospital can be beneficial for both the medical centre and the child, and it could result in reducing the overall cost of the procedure.

There are only a few studies on the safety of early feeding after PEG placement in paediatric patients. The recent prospective randomised study with the earliest feeding in children after PEG placement is by Corkins et al. [1]. Forty successive patients who met the entry criteria were enrolled, with 20 in each group, and they found no increase in complications with the earliest feeding (3 h after PEG insertion).

Our study will include a relatively large group of patients (100, 50 in each group), and the observation will last 12 months, which is longer than in other studies. First of all we would like to determine whether earlier initiation of feeds (3 h after PEG placement) is as effective and well tolerated as the current standard practice (8 h) and result in shortening the length of stay in hospital after PEG implantation. Both the objectives of the study and the primary, secondary and tertiary endpoints, are described in detail below. The goal is to establish an optimum standard procedure in the group of paediatric patients qualified for PEG insertion procedure.

## Methods

### Study design

This study is a multicentre, randomised, open label trial designed to evaluate the tolerance and safety of early enteral nutrition after PEG placement in children. Recruitment will be from patients attending one of six medical centres in Poland: The Department of Gastroenterology, Hepatology and Feeding Disorders and The Department of Paediatrics, Nutrition and Metabolic Disorders, The Children’s Memorial Health Institute in Warsaw, The Department of Paediatrics, Gastroenterology, Hepatology and Nutrition, The Medical University in Gdańsk, The Department of Allergology, Gastroenterology and Nutrition, The Medical University in Łódź, The Department of Paediatrics, The Medical University of Silesia in Katowice and The Department of Paediatrics and Gastroenterology, The Area Hospital in Toruń.

The trial will include 100 patients, from 1-month-olds to 18-year-olds, qualified for PEG placement. All participants will be prepared for the procedure according to the standard medical protocol.

Parents/legal guardians, and where possible also the patient, will be informed about the research plan, and after signed informed consent to participate in the study, patients will be enrolled on the study. At the Baseline visit participants will be randomised (1:1) to one of two treatment groups: Group I-the early enteral feeding group (3 h after PEG implantation) or Group II - the late enteral feeding group (8 h after PEG implantation). Follow-up visits in hospital are planned 3, 6, 9 and 12 months after the procedure in all subjects.

### Ethics

All the procedures are reviewed and approved by the Independent Review Board (Komisja Bioetyczna IP CZD, Approval Number: 73/KBE/2013). Patients and their caregivers will give their written informed consent before the start of any procedure.

The study will be conducted in accordance with the protocol, International Conference on Harmonization (ICH) guidelines, the applicable regulations and guidelines governing the conducting of clinical studies, and the ethical principles that have their origin in the Declaration of Helsinki.

The investigator or his/her representative will explain the nature of the study to the subject and the subject’s parent/legal guardian, and answer all questions regarding this study. The subjects will be included in all discussions in order to obtain verbal or written assent.

Prior to any study-related procedures being performed on the subject, the informed consent statement will be reviewed and signed and dated by the subject’s parent/legal guardian and the person who administered the informed consent.

### The selection of the study population

Subjects will be screened to determine whether they meet all the inclusion criteria specified below and have none of the exclusion criteria of this protocol.

The Inclusion Criteria are: age between 1 month and 18 years, medical indications for PEG placement, and informed consent to participate in the study signed and dated by the subject’s parent/legal guardian and also by the patient over the age of 16 years. Patients will be excluded if they: have serious, uncorrectable coagulation disorders, there is an inability to perform upper gastrointestinal (UGI) endoscopy (laryngeal or oesophageal stricture), or there is need for concomitant fundoplication or lack of technical ability to perform PEG placement procedure.

### Objectives

The main/primary objective of the study is to compare the tolerance of the early and late feeding modes after percutaneous gastrostomy placement in children.

The secondary objectives of the study are: to confirm the safety of early enteral nutrition after PEG implantation, to analyse the correlation between the feeding mode and the occurrence of early or late complications after the procedure and to compare the length of stay in hospital between two analysed groups.

Additionally, we would like to establish the impact of several factors (nutritional status, oropharyngeal flora, gastroesophageal reflux (GER), vitamin and trace element deficiency, faecal calprotectin level), on the complication rate and the PEG nutrition results. Apart from that we would like to establish the impact of PEG implantation on GER.

### Endpoints

The primary endpoint is the number of patients who will achieve full feed (total fluid and caloric requirements) within 48 h since the first feeding bolus.

The secondary endpoints are: the number of early complications (up to 6 days after PEG placement), the number of late complications (7 days-12 months after PEG placement), the duration of hospitalisation after PEG placement (in days), gastric residuals (ml) - total in the period up to 48 h since the first feeding bolus.

Tertiary endpoints are: improvements in nutritional status (3, 6, 9, 12 months after the procedure), body weight gain (kg) and height gain (cm), BMI kg/m^2^, the influence of vitamin and trace element deficiency, biochemical parameter abnormalities in the complications rate, the influence of ghrelin, leptin and adiponectin level on nutritional status improvement after PEG placement, the influence of gastroesophageal reflux (GER) diagnosed before the procedure on feeding tolerance, the correlation between the faecal calprotecine level and feeding tolerance after PEG placement, the complication rate and nutritional status before PEG placement, the influence of oropharymgeal flora on the complications rate (wound infection) and the correlation between PEG placement and GER.

### Randomisation

Subjects with clinical indications for percutaneous gastrostomy placement will be centrally randomised into one of the maintenance protocols: early enteral feeding-3 h after PEG placement (Group I) or late enteral feeding-8 h after PEG placement (Group II).

It was not possible to blind the nursing staff to the feeding time assigned to each patient.

### Intervention

All subjects will be prepared for the procedure according to established protocol, consistent with the actual ESPGHAN and ESPEN recommendations. Study procedures are listed below. Subjects will be fasted for 6 h before the procedure, where the child is only breast-fed, for 4 h. All subjects will receive one dose of intravenous antibiotic before the placement: Augmentin 30 mg/kg, in the case of hypersensitivity to beta lactam antibiotics: Clindamycin 3–6 mg/kg + Metronidazole 10 mg/kg. All the participants will receive primary gastrostomy button placement by the standard “pull” technique under general anaesthesia, using the Flocare PEG tube (Nutricia). Tissue samples from the stomach and duodemum will be obtained during the endoscopic examination for celiac disease screening. The severity of oesophagitis will be classified according to the Los Angeles Classification.

The procedure will be performed under general anaesthesia and afterwards patients will recover in a general post-anaesthesia-care area approximately for 2–3 h. Subjects will follow the same postoperative care, accordance with the standard established schedule. The postoperative procedures are introduced in Table 1.

**Table 1:** The postoperative procedures

First Day after procedure	• The first change of dressing - morning after PEG placement • The wound area is inspected (bleeding, erythema, secretion, induration, allergic skin reaction etc.), cleaned, disinfected and dried completely. • The tube should be pushed approximately 2–3 cm ventrally and carefully pulled back up to the resistance of the internal fixation flange. • Y-compress is applied under the tube • The external fixation plate is secured with free movement of at least 5 mm • Sterile dressing is applied
To 7 Days after the procedure	• Sterile dressing performed everyday • The wound area is inspected (bleeding, erythema, secretion, induration, allergic skin reaction etc.), cleaned, disinfected and dried completely.
7-14 Days after the procedure	• Sterile dressing performer every few days • The wound area is inspected (bleeding, erythema, secretion, induration, allergic skin reaction etc.), cleaned, disinfected and dried completely. • Washing with soap and water or showering is possible after initial wound healing

### Resumption of feeding

**Scheme 1:**
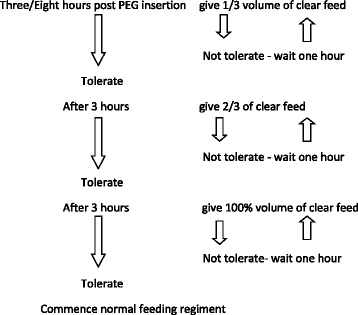
Feeding regimen after PEG insertion

The first feeding (Group I-3 h after the procedure, Group II-8 h after the procedure) will be with a polymeric diet (1 kcal/ml). The first feeding portion will have a volume equal to 1/3 of the full recommended portion, the volume of the second feeding will be equal to 2/3 of the full recommended portion and the third portion will be equal to the full recommended portion. Each portion will be introduced through the enteral feeding pump (Flocare Infinity, Nutricia), for 30 min, with a three-hour break between feeds (Scheme 1). The infusion of a 5 % glucose solution with electrolytes will be given through an intravenous line to cover the maintenance fluid requirements in all subjects.

### Study procedures

The study procedures are discussed in detail in this section. All study data will be recorded in documents. Subjects will be screened to ensure they meet all the inclusion criteria and none of the exclusion criteria. The patients and their caregivers will give their written informed consent before the start of any procedure. All subjects will undergo a radiological examination of upper gastrointestinal tract at screening. 24-h oesophageal pH monitoring/Multi-Channel Intraluminal Impedance and pH Measurement will be obtained at screening and after 10–12 months. A qualified physician will interpret the study results. Vital Signs-blood pressure, heart rate, respiratory rate, body temperature-will be monitored during hospitalisations. All measurements will be recorded in metric units when applicable. A physical examination, including weight and height, will be performed at each visit. Abnormalities noted will be evaluated and documented by the investigator as to whether they are complications. Oropharyngeal flora will be determined before PEG insertion at screening in all subjects. The microbiological flora will be examined in the event of wound infection in all subjects. Samples will be obtained for the laboratory tests. Clinical laboratory tests are listed in Table 2. All abnormal laboratory test results that are considered clinically significant by the investigator will be followed up to a satisfactory resolution. Plasma ghrelin, lepitin and adiponectin levels will be measured in all subjects after 72 h of enteral nutritional feeding (two measurements-fasting and 2 h after feeding). Test for faecal calprotectin level will be made in all subjects at screening. In all subjects the serum anti-tissue transglutaminase (tTG-IgA) and total IgA serum level will be measured for celiac-disease screening. Anthropometric parameters-height, weight, BMI, arm circumference, triceps skinfold and mean length of forearm-will be controlled during the study. Dietary consultation will be performed at screening and at control visits planned during the study. Complications will be monitored throughout the entire study period. Data on all study participants will be included in the safety analyses. At each visit, data about complications will be documented and blood samples will be obtained for laboratory evaluation. Any withdrawals from the study due to complications will be recorded in the study documentation. To evaluate parental/caregiver degree of satisfaction with the gastrostomy feeding the structured Satisfaction Questionnaire with Gastrostomy Feeding, the *SAGA-8* questionnaire, will be used 6 months after the PEG implantation. To investigate the influence of PEG placement on the family quality of life at Baseline Visit and 12 months after PEG implantation the validated *Family Quality of Life Questionnaire* ver. 2006 will be used.

**Table 2:** Clinical laboratory tests

Hematology	Clinical Chemistry	Coagulation
Hematocrit Hemoglobin Glycated hemoglobin Red Blood Cell (RBC) count White Blood Cell (WBC) count Neutrophils Bands Lymphocytes Monocytes Basophils Eosinophils Platelet count	Creatinine Urea Total protein Albumin Glucose Aspartate transaminze (AST) Alanine transaminaze (ALT) Sodium Potassium Calcium Zinc Iron Magnesium Selenium Ferritin C Reactive Protein Vitamin D – 25(OH)D3 Vitamin A Vitamin E Vitamin B 12 Ac folicum	Prothrombin time (PT/Quick) international normalized ratio (INR) activated partial thromboplastin time (APTT)

### Complications

Complications will be monitored throughout the entire study period. Data on all study participants will be included in the safety analyses. At each visit, complications will be documented and blood samples will be obtained for laboratory evaluation, and microbiological flora will be examined in the event of wound infection.

The occurrence of complications up to 6 days after PEG placement was considered as early complications. The occurrence of complications from 7 days to 12 months after the PEG placement was considered as late complications. Complications after PEG placement included: death < 72 h, vomiting, nausea, regurgitations, gastric residuals volume ≥ than in the previous feeding bolus (ml), GERD, aspiration pneumonia, diarrhoea, fever, bleeding from the gastrointestinal tract, local infection (reddening around the stoma canal, granulation tissue around the stoma, bleeding from the stoma canal, peristomal abcess), leakage of gastric contents, enlargement of the stoma canal, gastrointestinal cutaneous fistula, occlusion of the PEG tube, damage to the PEG tube, dislocation of the PEG system, buried bumper syndrome.

### Protocol deviations

The investigator should not implement any deviation from the protocol without prior review and agreement by Study supervision and in accordance with the Ethics Committee.

### Determination of sample size

Assuming the probability of an event in the control group of 0.6 and the probability of an event in the experimental group of 0.85, control per case subject 1, 0.8 statistical power, 0.05 alpha coefficient, the required size of each group was estimated at 50 (100 in total) with the Chi-square test.

### Planned methods of statistical analysis

The primary endpoints will include the ratio of patients who achieved full feed (liquid/calories) within 48 h of the first feeding bolus. The appropriate Chi-square tests will be used to compare the frequencies of primary endpoints, depending on the expected numbers. The intention to treat (ITT) analysis will include the determination of the proportions of patients with the primary endpoint achieved.

Secondary endpoints will include gastric residuals (total up to 48 h), the complications ratio, the duration of hospitalisation. The appropriate Chi-square tests for quality variables and the U Mann-Whitney test for quantity variables will be used.

Tertiary endpoints: the impact of nutritional status for PEG nutrition results: the appropriate Chi-square tests and uni- and multivariate analyses will be used.

Percutaneous endoscopic gastrostomy (PEG) was introduced for the first time in 1980 by Gauderer and Ponsky, and since that time the procedure has been modified and improved several times. PEG has become the preferred method for providing long term enteral nutrition in children with insufficient oral intake [2].

## Discussion

Percutaneous endoscopic gastrostomy (PEG) was introduced for the first time in 1980 by Gauderer and Ponsky, and since that time the procedure has been modified and improved several times. PEG has become the preferred method for providing long-term enteral nutrition in children with insufficient oral intake. The optimal timing for gastrostomy placement remains uncertain; it varies between 2 and 12 weeks of enteral feeding depending on recommendations [3–5]. According to the current ESPGHAN recommendations the anticipated duration of enteral nutrition exceeding 4–6 weeks [6] is an indication for gastrostomy and it can be prolonged in many cases. Before PEG placement each case should be considered on its own merits. the advantages and disadvantages must be assessed by a multidisciplinary nutrition-support team, taking into account clinical condition, diagnosis, prognosis, ethical issues, patients’ and parents’ expectations and expected effect on the quality of the child’s life. In general, PEG can be used as a means of exclusive or supplemental enteral tube feeding, gastric decompression and/or the administration of medications [7]. It can significantly reduce feeding time, improve nutritional status and growth, and also the social functioning or quality of life [8, 9]. The range of indications for PEG tube use is wide and has been demonstrated in children with neurodisability, congenital heart disease, cystic fibrosis, neonatal pulmonary disease, oncological disorders, metabolic disease, genetic-chromosomal and degenerative disease, Crohn’s disease and chronic renal failure [10]. The former indication for PEG placement is dysphagia associated with neurological or neuromuscular disorders, especially cerebral palsy [11].

The appropriate time to initiate enteral nutrition after the placement of a percutaneous endoscopic gastrostomy (PEG) tube has been an area of limited research. Historically, data to guide decisions regarding the initiation of feedings has been extrapolated from surgical experience with gastrostomy tubes. The recommended time for the resumption of feeding in adults and children is inconsistent, and varies from 1 to 24 h [5]. There are few published trials in the literature to guide post-PEG feeding decisions. Choudhry et al. prospectively analysed 41 subjects in a randomised controlled fashion to early (after 3 h) versus delayed feedings (after 24 h) after PEG placement [12]. Two subjects in the early intervention group versus one subject in the late intervention group had feedings held due to significant gastric residuals (arbitrarily defined significant gastric residual volumes as 60 mL). Complication rates and mortality data were similar among the groups. Stein et al. prospectively enrolled 80 patients in a randomised controlled trial comparing immediate feeding within one hour after PEG placement versus feeding after 24 h in intensive care and intermediate unit patients [13]. Gastric residual volumes were measured as the primary endpoint. Residual volumes were similar between groups on days 1 and 3. Complication rates and mortality rates were similar between groups. Brown et al. prospectively enrolled 57 patients in a controlled randomised trial comparing early (within 3 h) versus a delayed (next-day) feeding regimen post PEG-placement [14]. All patients tolerated the feeding regimen without complication. Chumley et al. describes in abstract form a prospective randomised controlled study of 150 patients [15]. Three groups were compared. Groups 1 and 2 were fed early (Group 1, *n* = 50 — fed at 3 h; Group 2, *n* = 50 — fed at 6 h). Delayed feedings took place after 24 h. Only one patient in the 3-h group developed increased gastric residuals (volume not defined). McCarter et al. prospectively studied 112 patients in a controlled randomised fashion comparing early feedings (4 h) versus delayed feeding (24 h) after PEG placement [16]. Twenty five percent of the early feeding group had a high gastric residual on day 1 compared to 9 % in the delayed feeding group (*p* = <0.029). By day two, there was not a statistically significant difference in gastric residuals between the groups. One patient in the study died of aspiration pneumonia (delayed-feeding group). Minor complications were similar between the groups. In 2008 Bechtold et al. performed meta-analysis and only RCTs on adult subjects that compared early (≤4 h) versus delayed or next-day feedings after PEG placement were included [17]. Authors concluded that early feeding can represent a safe alternative to delayed or next-day feedings. Although an increase in significant gastric residual volumes at day 1 was noted, overall complications were not affected. Szary et al. in 2011 in their meta-analysis demonstrated that feeding initiated 3 h after PEG placement in adult patients is safe and effective [18]. By initiating feeding earlier after PEG placement, many patients can avoid acute care hospital stays, unnecessarily prolonged intravenous access and medications and receive earlier substantial nutrition. The study evaluating the safety of early (6 h) feeding in children after PEG placement (6 h) was performed by Werlin et al. [19]. This was an uncontrolled series of 24 paediatric patients, and they found no increase in the complication rate with the earlier feedings. The recent prospective randomised study with earliest feeding in children after PEG placement is by Corkins et al. [1]. Forty successive patients who met the entry criteria were enrolled, with 20 in each group. The authors compared the tolerance of feedings at 3 and 6 h after percutaneous endoscopic gastrostomy placement. The change in abdominal girth before and 1 h after the initial feeding, any vomiting, and the gastric residual volume before the next feeding. were recorded and the length of stay was also documented. They found no increase in complications with the earliest feeding (3 h after PEG insertion).

Concluding, there aren’t sufficient randomised prospective controlled trials in the paediatric population comparing the safety and tolerance of early feeding after PEG placement (3–4 h). Most patients are fasted for at least 12 h following percutaneous endoscopic gastrostomy. In order to decrease the period of fasting, inadequate nutritional support, and hospitalisation time, we decided to design this study. The additional goal is to establish an optimum standard procedure in the group of paediatric patients qualified for PEG insertion procedure in Poland.
